# Novel Chitinase Gene *LOC_Os11g47510* from Indica Rice Tetep Provides Enhanced Resistance against Sheath Blight Pathogen *Rhizoctonia solani* in Rice

**DOI:** 10.3389/fpls.2017.00596

**Published:** 2017-04-25

**Authors:** Kamboj Richa, Ila M. Tiwari, B. N. Devanna, Jose R. Botella, Vinay Sharma, Tilak R. Sharma

**Affiliations:** ^1^National Research Centre on Plant BiotechnologyNew Delhi, India; ^2^Department of Bioscience and Biotechnology, Banasthali UniversityBanasthali, India; ^3^School of Agriculture and Food Sciences, The University of Queensland, St LuciaQLD, Australia; ^4^National Agri-Food Biotechnology InstituteMohali, India

**Keywords:** chitinase, rice, *Rhizoctonia solani*, sheath blight, QTL, Tetep, transgenic

## Abstract

Sheath blight disease (ShB), caused by the fungus *Rhizoctonia solani* Kühn, is one of the most destructive diseases of rice (*Oryza sativa* L.), causing substantial yield loss in rice. In the present study, a novel rice chitinase gene, *LOC_Os11g47510* was cloned from QTL region of *R. solani* tolerant rice line Tetep and used for functional validation by genetic transformation of ShB susceptible japonica rice line Taipei 309 (TP309). The transformants were characterized using molecular and functional approaches. Molecular analysis by PCR using a set of primers specific to CaMv 35S promoter, chitinase and *HptII* genes confirmed the presence of transgene in transgenic plants which was further validated by Southern hybridization. Further, qRT-PCR analysis of transgenic plants showed good correlation between transgene expression and the level of sheath blight resistance among transformants. Functional complementation assays confirmed the effectiveness of the chitinase mediated resistance in all the transgenic TP309 plants with varying levels of enhanced resistance against *R. solani*. Therefore, the novel chitinase gene cloned and characterized in the present study from the QTL region of rice will be of significant use in molecular plant breeding program for developing sheath blight resistance in rice.

## Introduction

Sheath blight caused by *Rhizoctonia solani* is a major constraint in rice cultivation. Yield losses due to sheath blight infection ranges from 8 to 50% depending on severity of the disease, stage of the crop at which it was infected by the fungus and overall environmental conditions (Savary et al., 2000; Singh et al., 2004). To withstand these kinds of stresses, plant have evolved a battery of self-defense systems (Datta et al., 2001) among the different self-defense mechanisms, those mediated by the resistance (*R*)-genes and other defense response (DR) genes are most significant. Upon interaction with any pathogen, plants activate their self-defense system leading to production of an array of pathogenesis related (PR) proteins like chitinases, glucanases, thaumatin-like proteins etc.

Till date, absolute resistance to *R. solani* has not been identified in any crop. Its broad host specificity and saprophytic nature renders traditional control techniques like crop-rotation inefficient. Due to lack of inherent resistance against ShB, disease control is troublesome and extensive use of chemicals remains the main strategy of disease control. However, chemical control contributes to increase health risk and financial strain on the farmer as well as being disastrous to the environment. In the absence of natural resistance, genetically modifying plants with transgenes for enhanced resistance is the most suitable alternative (Lin et al., 1995; Datta et al., 2000, 2001; Kim et al., 2003). Antifungal proteins such as chitinases and glucanases that can hydrolyse the chitin and glucan components of the fungal cell wall are the popular choices (Okubara et al., 2014).

Chitinases are the members of PR-3 group of PR proteins responsible for hydrolysis of chitin, a structural polysaccharide of the cell wall of many fungi, yeast, insects, etc. Chitinases were first described by Bernard in orchid bulb as a thermosensitive and diffusible antifungal factor (Sharma et al., 2011). Among the other sources, microbial chitinases are the major and most preferred, due to their availability and rapid multiplication. Besides microbes, chitinases are also reported in plants, animals and human beings. Chitinases purified from plants, microbes, and animals show a strong antifungal activity *in vitro* (Neuhaus, 1999). Based on the amino acid sequences, plant chitinases have been classified into five different classes. Class I, II, and IV belongs to chitinases family 19 and contains globular domains whereas Class III and class V belongs to the chitinases family 18 and have 8 α-helices and β-strands each as key structures (Fukamizo et al., 2003; Hamid et al., 2013). Chitinases have received a wide attention toward the biocontrol of fungal phytopathogens, due to their direct role in chitin degradation. Till-date, many chitinase genes have been identified, cloned and transformed into different plants in order to provide resistance against various plant diseases. Sundheim et al. (1988) cloned chitinase genes from *Serratia marcescens* and overexpressed them in *Pseudomonas fluorescens*. The transconjugant *P. fluorescence* successfully inhibited the growth of *Fusarium oxysporum*, resulting in reduced infection of radish plants by same pathogen. Chernin et al. (1997) used recombinant *Escherichia coli* expressing chitinase gene of *Enterobacter agglomerans* in rhizosphere which decreases the onset of *R. solani* infection in cotton. Subsequently, these chitinase genes were directly deployed in plants, through transgenic approach. Datta et al. (2001) demonstrated effectiveness of chitinases against *R. solani* and *Magnaporthe grisea* in rice. Basic PR-3-type chitinase from bean when overexpressed in transgenic tobacco and canola successfully suppressed the infection caused by *R. solani* (Grover and Gowthaman, 2003). Overexpression of rice chitinases has shown resistance against *Uncinula necator* in grapevine, *Botrytis cinerea* in cucumber, *Puccinia coronata* in chrysanthemum and Italian ryegrass, *Mycosphaerella fijiensis* in banana and Fusarium wilt and early blight in tomato (Tabei et al., 1998; Takatsu et al., 1999; Yamamoto et al., 2000; Kishimoto et al., 2002; Takahashi et al., 2005; Kovács et al., 2013; Jabeen et al., 2015).

Previously, using QTL mapping approach, we identified and mapped a major sheath blight resistance QTL: qSBR11-1 on chromosome 11 of resistant rice line Tetep (Channamallikarjuna et al., 2010). Subsequently we deciphered this locus and characterized 11 novel chitinase genes present in this QTL region (Richa et al., 2016). It was found that among the 11 chitinase genes present in this particular QTL, expression of *LOC_Os11g47510* was much higher than rest of the genes at 36 hours post inoculation (hpi) with *R. solani*. Biochemical assays also confirmed antifungal activity of this particular gene against the rice sheath blight fungus *R. solani*. Further the field resistance of these genes against sheath blight pathogen was reported by Singh et al. (2012). They transferred QTL qSBR11-1 from Tetep into indica rice line Improved Pusa Basmati 1 and proved that qSBR11-1 imparts a significant level of field resistance against *R. solani*. However, there is no report on the dissection of QTL region at gene level and its validation in case of *R. solani*-rice system. The present study was thus designed to know the *in planta* expression and resistance response of highly induced gene *LOC_Os11g47510* from this QTL in susceptible rice cultivar Taipei 309. In the present study, we report the potential application of rice chitinase gene cloned from a tolerant rice line Tetep to enhance the resistance against sheath blight disease (ShB) in susceptible rice as well as the usefulness of the detached leaf bioassay for initial screening of the putative transformants for ShB resistance.

## Materials and Methods

### Plant Materials and Candidate Gene

Seeds of rice line Taipei 309 (TP309), susceptible to *R. solani* were available with the authors. *Loc_Os11g47510*, rice chitinase gene showing highest expression among other chitinase genes of the QTL qSBR11-1 was validated by qRT-PCR analysis after infection with *R. solani* and displayed antifungal activity during bioassay was previously cloned and reported by the authors (Richa et al., 2016).

### Development of *Loc_Os11g47510* Gene Construct for Plant Transformation

Two different set of primers specific to *Loc_Os11g47510* (from Tetep and TP309) and *HptII* gene were designed using Primer 3 primer designing tool (Supplementary Table 1). Primers specific for *LOC_Os11g47510* gene had sequence of restriction sites *Bam*HI and *Xba*I in the forward and reverse primers, respectively, whereas those for *HptII* gene had *Sac*II restriction enzyme sites in both the forward and reverse primers. *LOC_Os11g47510* of Tetep was amplified from a clone which was already confirmed by PCR and restriction digestion (Richa et al., 2016), *LOC_Os11g47510* allele from TP309 was also amplified using same set of primers whereas *HptII* gene was PCR amplified from pCAMBIA1305.1. *LOC_Os11g47510* was cloned at corresponding sites in pRT100 vector and confirmed. *LOC_Os11g47510* allele from TP309 was cloned in pJET1.2 vector and sequence confirmed. The *HptII* gene was cloned into the *Sac*II site of the pBS(SK+) vector and clones were confirmed by PCR and restriction digestion. Subsequently, the final construct for rice transformation was developed by cloning the entire PCR amplified fragment (CaMv35S + *Loc_Os11g47510* + *nos* terminator) using primers specific to 35S promoter and *nos* terminator having *Sma*I restriction enzyme sites in both forward and reverse primers from pRT100 vector. This fragment was cloned at the corresponding restriction enzyme sites in modified pBS(SK+) vector (already having *HptII* gene). PCR reaction was carried out by using Finnzymes Phusion^®^ High-fidelity DNA polymerase using standard protocol from manufacturer (Thermo Scientific) (Supplementary Figures 1, 2). The *LOC_Os11g47510* DNA sequences from Tetep and TP309 were aligned using MULTALIN tool^1^ (Supplementary Figure 3).

### Callus Induction in Rice Line TP309

Mature seeds of TP309 were dehusked manually and thoroughly washed with Teepol detergent followed by three washes with sterilized distil water. Sterilization of the seeds was performed by following method: seeds were dipped in 70% ethanol and gently stirred for 1 min followed by three washing with sterile water. Then 5% sodium hypochlorite treatment was given for 20 min and washing was given thrice with sterile distilled water. Finally 10 min sterilization with 0.2% HgCl_2_ was done and traces of HgCl_2_ was removed by washing with distilled water for five times. Sterile seeds were then drained free of water and blotted dried on a filter paper. The sterilized seeds were inoculated on callus induction media (CIM); Murashige and Skoog (MS) medium containing sucrose (30.0 g/l) + proline (500 mg/l) + casein hydrolysate (400 mg/l) + 2,4-D (2 mg/l) + agar (8.0 g/l) by placing 25 seeds per plate and incubated in dark at 28°C. Twenty-days-old embryogenic calli were excised and about 30 of them were closely arranged in a circle at the center of petriplate containing osmoticum medium (MS + 30.0 g/l sucrose + 0.2 M mannitol + 0.2 M sorbitol) supplemented with agar (8.0 g/l). The calli were ready for bombardment after 4 h of osmoticum treatment.

### Genetic Transformation of TP309

TP309 calli were transformed using biolistic approach (Sanford et al., 1987). Intact plasmid constructs were bombarded using helium driven particle delivery system (PDS 1000, Bio-Rad). DNA were precipitated and coated to gold particles following the procedure recommended by Sanford et al. (1987). The calli were placed in the center of petridish containing osmotic medium (MS media with 8% bacteriological agar, 30 g/l mannitol and 30 g/l sorbitol). These calli were bombarded with the DNA-coated gold particles and were then maintained at 28°C under dark condition. The distance from the stopping screen to target was 9 cm, and the rupture disk strength was 1100 psi under a vacuum of 25 mm of Hg. After 16 h of bombardment, calli were transferred to the selection medium; CIM + Hygromycin (50 mg/l) and incubated in dark at 28°C. After 15 days, the proliferating calli were again transferred to the fresh selection medium for two more rounds of selection of 15 days each. After a total of 45 days of selection, healthy and proliferating calli were transferred to the regeneration media.

### Plant Regeneration

The calli survived after three round of selection in hygromycin containing medium were transferred to the regeneration medium [MS + BAP (2.5 mg/l) + NAA (1.5 mg/l) + agar (8 g/l) + hygromycin (50 mg/l)] and incubated at 28°C under 1000 lux with 16 h light and 8 h dark regime. Entire process of selection and regeneration took about 2–3 months for completion. As the plantlets reached 2 to 3 inch of height with well developed roots, they were acclimatized in solarite and finally transferred to the National Phytotron Facility (NPF), IARI, New Delhi, India and maintained under standard conditions required for rice.

### Molecular Analysis of the T0 and T1 Plants

The transgenic plants obtained from the present study were validated using molecular biology tools like PCR, qRT-PCR and Southern blot hybridization. To determine the presence of the transgene, genomic DNA was extracted from leaf tissues of transgenic plants and analyzed using PCR. For PCR validation, one set of primers specific to promoter and *LOC_Os11g47510* gene and another set specific for *HptII* gene were used. The details of different primers used and the annealing temperature used for PCR are given in Supplementary Table 1. For PCR, DNA was denatured at 94°C for 3 min, followed by 35 cycles of amplification [45 s at 94°C; 30 s at 58°C; 45 s at 72°C] and final incubation at 72°C for 10 min. For gene integration analysis, we performed southern hybridization. High quality genomic DNA was isolated by CTAB method. 10 μg gDNA was completely digested with *Eco*RI restriction enzyme. Digested DNA samples along with positive control were electrophoresed on 0.8% agarose gel in 1× TBE buffer. After electrophoresis, DNA samples were denatured and transferred on to the nylon membrane. 192 bp fragment of the *HptII* gene was labeled with α-dUTP and used as a probe for hybridization. After that, southern blotting was performed by using standard protocol (Sambrook et al., 1989).

Real-time quantitative Reverse Transcription PCR (qRT-PCR) analysis was performed to analyze the relative expression of the integrated chitinase gene as well as *hptII* gene in control and transgenic TP309 plants, separately. Total RNA was isolated from the leaf samples of control and transgenic leaves using RNeasy Plant Mini Kit (Qaizen, USA). Quality and quantity of RNA was analyzed by agarose gel (1.0%) electrophoresis and nanodrop (Thermo Scientific), respectively. cDNA was prepared from 1 μg of each of total RNA sample using Protoscript first strand cDNA synthesis kit (New England Biolabs, UK). 18srRNA primer was used as internal control to normalize the data. Specific primers were designed for *LOC_Os11g47510, hptII* and 18srRNA gene and used for qRT-PCR (Supplementary Table 1). cDNA prepared was used as a template for expression analysis by Light cycler SYBER green I master Kit using Light Cycler^®^ 480 II PCR system (Roche) using manufacturer’s guidelines. The qRT-PCR cycling conditions were: initial denaturation 95°C; 5 min, 50 cycles of 95°C; 10 s, and annealing temperature 60°C; 15 s, 72°C; 15 s.

### Functional Characterization of Transgenics in Response to *Rhizoctonia solani* Infection

Detached leaf bioassay was performed in control and transgenic plants to check phenotypic response of these plants mediated by the candidate gene against *R. solani*. For detached leaf assay, mycelium of *R. solani* was inoculated on PDA plates. Equal sized agar plugs containing mycelium of *R. solani* were inoculated on the surface of detached leaves (transgenic and control plant) and they were then placed on the moist filter paper in the petriplates. Petriplates were then sealed with parafilm to retain the moisture inside the plates. Phenotypic observation was recorded as degree of infection of *R. solani* at different hpi. Statistical analysis was performed for the three replicates of each of the transgenic plant.

### Pathogenesis of *R. solani* on Transgenic Rice Leaves

Microscopic study was performed to study the growth of *R. solani* mycelium in transgenic as well as NT-TP309 leaves. Leaves were placed over wet filter paper in the petriplates and inoculated with agar plugs containing *R. solani* mycelia. At 24, 48 and 72 hpi, leaves were fixed on filter papers soaked with 1:1 (v/v) Ethanol: Acetic acid for 24 h and later transferred on to filter paper soaked in lacto glycerol [1:1:1 (v/v) ratio of lactic acid:glycerol:water). Samples were then stained with aniline blue (0.1% w/v) in 0.1 M K3PO4 and excess dye was removed using 0.1 M K3PO4. Leaves were mounted on to microscopic slides in 10% glycerol and observed under light microscope.

### Quantification of Fungal Biomass

Standard calibration curves were established to measure the fungal biomass in the *R. solani* inoculated samples in T_1_ generation. Pure *R. solani* fungal gDNA was used for serial dilutions to obtain 0.002, 0.02, 0.2, 2 and 20 ng/μl of gDNA. pUC19 plasmid DNA (Thermo Fisher Scientific) was also diluted to obtain 0.001, 0.01, 0.1, 1, and 10 ng/μl DNA. These diluted DNA were mixed separately with a constant amount of 10 ng of genomic DNA isolated from uninfected TP309 leaves. Calibration curves were generated by plotting CT values (averaged from three technical repeats) obtained by qRT-PCR against the corresponding logarithmic *R. solani* and pUC19 DNA amounts. For the absolute quantification of fungal DNA in inoculated leaf samples, 20 ng/μl genomic DNA was used as a template. Two qPCR reactions were run separately by using the (*Rhizoctonia* Map Kinase gene) RPMK1_F and RPMK1_R and M13-F and M13-R pair of primers. CT values obtained were then used to calculate the absolute amount of target DNA in a given reaction. For data normalization, 0.1 ng of pUC19 DNA was mixed with all the transgenic and NT plants DNA.

## Results

### Development of Plant Transformation Vector

**FIGURE 1:**
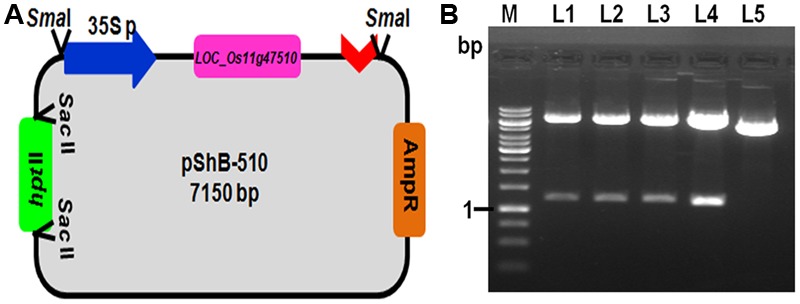
**Development of plant transformation gene cassette containing *LOC_Os11g47510.* (A)** Schematic representation of the pShB-510 vector. **(B)** Restriction digestion of the putative positive clones with *Bam*HI restriction enzyme. Lane L1–L4- putative positive clones; Lane L5- Empty pBS (SK+) vector containing hygromycin gene; Lane M- 1 kb DNA Ladder.

Chitinase gene from rice line Tetep, present on the locus *LOC_Os11g47510*, which was showing higher relative expression in qRT-PCR as well as highest chitinase activity in *in vitro* expression study was selected for plant transformation experiments (Richa et al., 2016). The putative recombinant pBS(SK+) plasmids with *LOC_Os11g47510* gene cassette (CaMv 35S promoter + *LOC_Os11g47510* gene+ nos terminator) were screened for gene integration by PCR using primers specific to this region and also by restriction digestion using *Bam*HI (**Figure 1**). The PCR reaction (1.6 kb promoter and gene fragment along with nos terminator) and restriction digestion confirmed (∼1.2 kb gene fragment) the presence of gene cassette and gene, respectively (**Figure 1** and Supplementary Figure 2). This particular gene cassette was cloned in a modified pBS(SK+) vector which is having *hptII* gene for plant selection and this modified plant transformation vector was used for genetic transformation experiments and referred to as pShB-510.

### Genetic Transformation of TP309 Rice Line with the Chitinase Gene

Genetic transformation of rice line TP309 was performed with gene cassette containing DR gene *Loc_Os11g47510* using biolistic approach. For transformation, healthy calli of TP309 were selected and used for bombardment using particle gun. Sixteen hour post bombardment, the calli were transferred to the selection media containing 50 mg/l hygromycin. At the end of three selection cycles of 15 days each, out of 428 and 400 calli initially used for bombardment in two different set (batches) of experiments, 148 and 112 transformed calli, respectively, were obtained. The proliferating, healthy calli were then transferred to the regeneration medium supplemented with combination of auxin and cytokinin (NAA & BAP) along with hygromycin for selection (Supplementary Figure 4). Successfully regenerated plants from individual calli from each experiment were treated as an event and subjected to hardening. Finally 51 plants from nine events were transferred to the pots and grown in National Phytotron Facility (NPF, IARI, New Delhi, India) and seeds were obtained from eight out of the nine events. Regeneration efficiency was determined for different batches by calculating the percentage of regenerated calli from total number of calli subjected to regeneration after third selection cycle. The overall regeneration frequency was found to be around 55.3%, however, overall transformation efficiency achieved was 6.25% which was validated by molecular analysis (Supplementary Table 2).

### Molecular Characterization and Functional Validation of Putative Transgenic Plants

**FIGURE 2:**
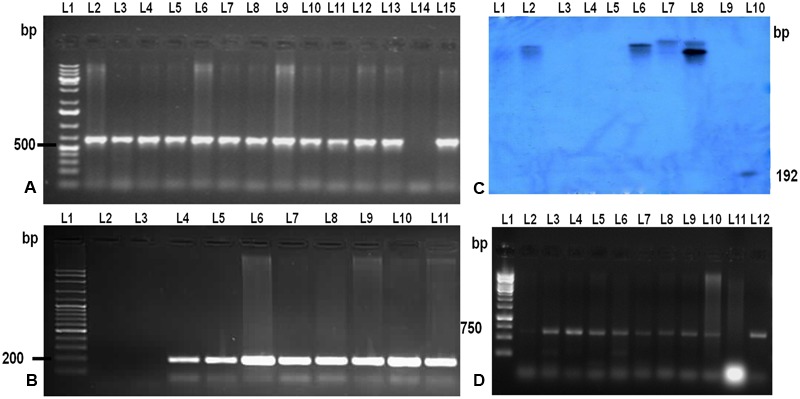
**Molecular analysis of the T0 and T1 transgenic TP309 plants. (A)** PCR analysis of putative T0 transgenic with CaMv 35S promoter forward primer and reverse primer from the middle of the gene. Lane 1, 1 kb plus DNA ladder; Lane 2–13, putative transformants; Lane 14, –ve Control; Lane 15, +ve control. **(B)** PCR analysis of putative transgenic with hygromycin specific primers. Lane 1, 100 bp plus DNA ladder; Lane 2, –ve control; Lane 3–10, putative transformants; Lane 11, +ve Control. **(C)** Southern Blotting of transgenics. Lane 1, λ digested DNA with *Hind*III; Lane 2–8, putative transformants; Lane 9, –ve control; Lane 10, +ve control (192 bp Probe). **(D)** PCR analysis of T1 transgenic with CaMv 35S promoter forward primer and reverse primer from the middle of the gene. Lane 1, 1 kb DNA ladder; Lane 2–10, T1 transgenics; Lane 11, –ve control;; Lane 12, +ve control.

To confirm the presence and integration of the candidate gene, *LOC_Os11g47510* in putative transgenic plants, PCR and Southern blot hybridization was performed using genomic DNA isolated from healthy and normal looking putative transgenic plants (**Figures 2A,B,C**). PCR amplification using two primer sets: one set specific to *hptII* and another set specific to promoter region and transgene was performed. PCR results revealed that out of 35 plants, only 12 plants belonging to eight events were positive for both transgene and *hptII* gene.

Further, copy number of the integrated gene *LOC_Os11g47510* was analyzed by Southern hybridization in all eight PCR positive putative transformed rice plants (**Figure 2C**). Hybridization was performed using DIG labeled *HptII* specific DNA probe in this study. Single and double insertions were observed in the putative transgenics and no insertion was found in the non-transgenic TP309 (NT) plant confirming the integration of the transgene in transgenics. Different positioning of the transgene in the autoradiogram confirmed that they are independent events. T1 seeds of the putative transformants were screened on hygromycin containing media and raised in the phytotron. Their transgenic nature was also confirmed by molecular analysis (**Figure 2D**).

### Expression Analysis of *LOC_Os11g47510* and *hptII* Gene in Transgenic Plants

**FIGURE 3:**
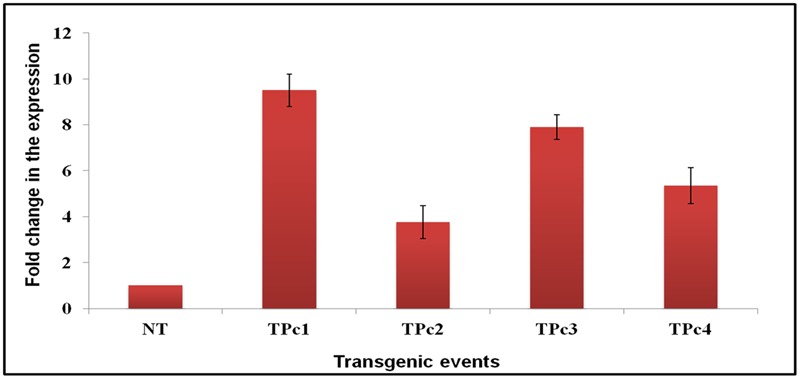
**Differential expression analysis of *LOC_Os11g47510* gene in transgenic vs. control TP309 plants.** Leaf samples of southern positive transgenic plants and non-transgenic plant were subjected to qRT-PCR for expression study of gene under CaMv 35S promoter. qRT-PCR analysis indicates that *LOC_Os11g47510* gene is expressing constitutively and showing different expression levels in different transgenic events. NT, Non-transgenic plant, TPc1–Tpc4, Transgenic events.

Expression study of the gene *LOC_Os11g47510* in transgenic plants was performed using qRT-PCR technique. All four Southern blot positive transgenic plants along with control (NT) TP309 plant were screened with the primers specific to *Loc_Os11g47510* transcribed region. 18S rRNA gene specific primers were used as an internal control in the present experiment for data normalization. The results obtained indicated varying level of transgene expression, ranging from 3.7- to 9.5-folds in different transgenic events (**Figure 3**). The expression of *hptII* in NT and transgenic plants revealed no amplification in NT-TP309, but varying levels of expression of four transgenic lines (Supplementary Figure 5).

### Functional Analysis of Transgenic Plants by Detached Leaf Bioassay

**FIGURE 4:**
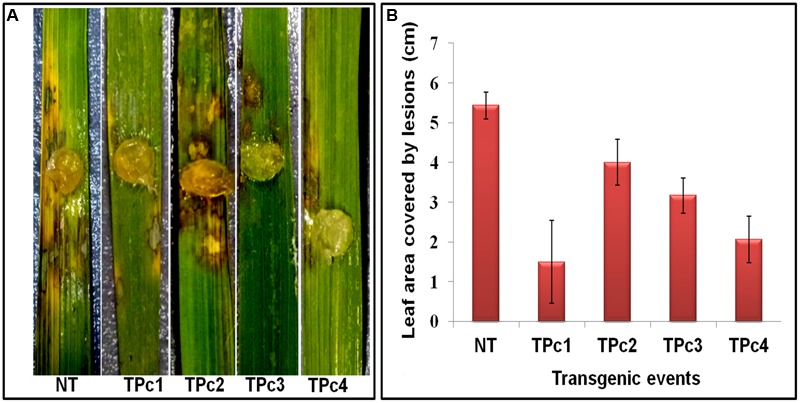
**Detached leaf Bioassay of control TP309 vs. transgenics plants. (A)** Detached leaves of transgenic and non-transgenic plants were placed on the wet filter paper. Agar plug containing the mycelium of *R. solani* were placed on the surface of the leaves. Petriplates were sealed with parafilm and incubated at 28°C for 3 days. **(B)** Statistical analysis of lesion covered leaf area (after 72 h) was performed by calculating the standard error of mean of the three replicates for each of the plant. NT, Non-transgenic plant, TPc1–TPc4, Transgenic events.

Detached leaf bioassay experiments using transgenic and NT-TP309 plants confirmed the transgene mediated resistance response in transgenic plants in comparison to NT plants (**Figure 4** and Supplementary Figure 6). At 36 hpi, size of the lesions developed on transgenics was comparatively smaller than control NT-TP309 plants. After 72 hpi, almost whole leaf of NT-plant was infected with sheath blight pathogen *R. solani*, whereas the infection on the transgenics was restricted to small lesions.

### Microscopic Study of Transgenic Rice Leaves for Fungal Growth Analysis

**FIGURE 5:**
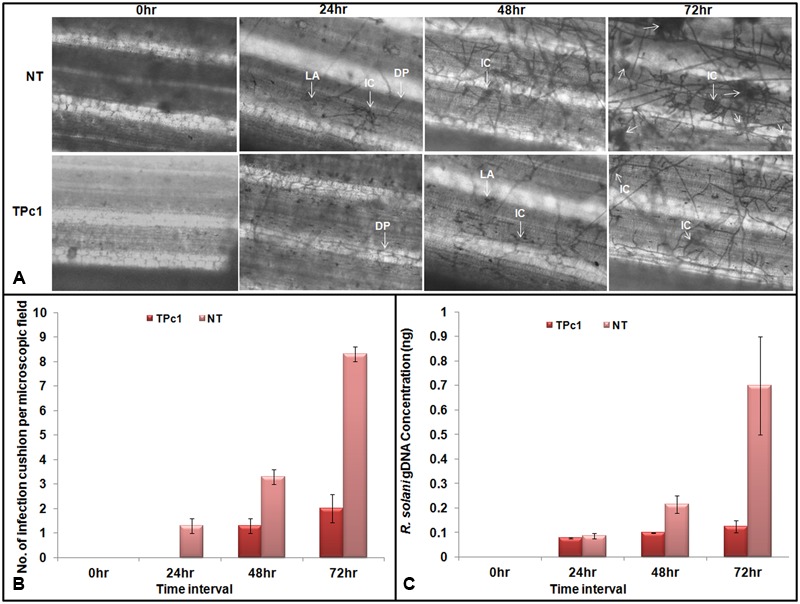
**Microscopic study of fungal mycelium inside the rice leaves. (A)** Microscopic observations of lesions in infected transgenic and NT rice leaves at 24, 48, and 72 hpi. Mycelia of *R. solani* grew and colonized surface of the leaves. Post colonization, branching of fungal hyphae was observed which either directly penetrate the host cells or form a lobate appressorium and invaded leaf tissue via epidermal intercellular spaces. The number of infection cushion observed in non-transgenic leaves at 24, 48, and 72 hpi was higher in NT-TP309 plants than transgenic lines. **(B)** Number of infection cushion per microscopic field observed under the microscope in NT and transgenic plants at different time intervals. **(C)** Fungal biomass quantification. qPCR analysis using DNA from detached leaf samples of transgenic as well as non-transgenic plants inoculated with *R. solani*. Amount of fungal DNA increases with increase time interval after inoculation in both transgenic as well as non-transgenic leaves but rate of increase was much higher in NT rice leaves as compare to the transgenic leaf samples. Statistical analysis for each experiment was performed by calculating the standard error of mean of three replicates. DP, Direct penetration, LA, Lobate appressorium, IC, Infection cushion.

Observations of *R. solani* infected leaves revealed that lesion development begins at 24 hpi in NT-TP309 leaves whereas no lesion was observed in transgenic TP309 leaves. Mycelia of *R. solani* grew and colonized surface of the leaves in both transgenic and NT plants. After colonization, hyphal branches were seen and they either penetrated rice cells directly or formed lobate appressorium and invaded leaf tissue via epidermal intercellular spaces as well as through stomata and trichomes (**Figure 5A**). Usually, *R. solani* hyphae tends to grow in bunch and ultimately form infection cushions. Similarly we observed penetration peg or infection cushion in NT leaves at 24 hpi, and after 24 hpi number of infection cushions increased with increase in time (at 48 and 72 hpi), whereas in case of transgenic rice leaves infection cushion was observed only at 72 hpi (Supplementary Figure 6). The size of the hyphal mat at the site of colonization and also lesion size was comparatively less in the transgenic lines. We also recorded the number of infection cushions per microscopic field in both transgenic and NT leaves at each time interval. At 72 hpi, the number of infection cushions per microscopic field in transgenic plant leaves was only 2 as compared to 8.3 in NT plant leaves (**Figure 5B**).

### Quantification of Fungal Biomass in the Infected Tissues

Absolute quantification of the fungal biomass in case of transgenic as well as NT rice leaves inoculated with *R. solani* was analyzed by using qPCR. The results obtained revealed that with progress in time interval, the amount of the fungal DNA has increased in both NT as well as in transgenic leaves; however, the amount of fungal DNA found in the case of NT leaves was much higher than the amount in transgenic plants (**Figure 5C** and Supplementary Figure 7).

## Discussion

Rice ShB is one of the most destructive diseases causing severe yield losses all over the world (Lee and Rush, 1983). Management of rice sheath blight is difficult as *R. solani*; the causal agent of the disease is having a broad host range (Taheri et al., 2007). Further, breeding for sheath blight resistance is very tedious task and time consuming, largely due to quantitative nature of the resistance response. However, now-a-days, cloning of the genes responsible for resistance into the susceptible rice varieties is gaining popularity over other approaches. The developments in the field of identification and mapping of resistance genes and the knowledge of signal transduction components involved in hypersensitive response and systemic acquired resistance pathway had increased the available sources for developing disease resistant plants (Piquerez et al., 2014). Previous studies have proved that the constitutive expression of PR genes has increased resistance against plant pathogens in transgenic plants (Xiong and Yang, 2003; Sayari et al., 2014).

Till date many genes had been cloned and transformed into the susceptible varieties against the fungal diseases. Among the cloning of disease resistance genes, overexpression of gene from the same plant species instead of foreign transgene is advantageous in consideration to public acceptance (Datta et al., 1999). Alexander et al. (1993) demonstrated the overexpression of PR-1a gene in tobacco has increased the tolerance against *Peronospora tabacina* and *Phytophthora parasitica* var. *nicotianae*. Thaumatin like PR-5 overexpressed in transgenic rice and orange has increased resistance against *R. solani* and *P. citrophthora*, respectively (Datta et al., 1999; Fagoaga et al., 2001). PR-3 specific β-1,3-glucanases from alfalfa, barley, tobacco and soybean have been shown to suppress fungal diseases in tobacco (Bucher et al., 2001). Overexpression of glucanases from soybean has improved tolerance of potato and kiwi to *P. infestans* and *B. cinerea* infection, respectively (Grover and Gowthaman, 2003), whereas a glucanase of potato was reported to increase resistance in flax against *Fusarium oxysporum* and *F. culmorum* (Wrobel-Kwiatkowska et al., 2004).

Huynh et al. (1992) demonstrated the *in vitro* antifungal activity of 28 kDa chitinases (*chiA* and *chiB*) isolated from maize seeds against the growth of *Trichoderma reesei, Alternaria solani* and *Fusarium oxysporum.* An antifungal chitinase of 64 kDa from *Enterobacter* sp. NRG4 against *R. solani* was reported by Dahiya et al. (2005).

In previous study we cloned and characterized 6 out of 11chitinase genes present in the QTL qSBR11-1 from rice line Tetep and performed expression analysis of those 11 chitinase genes present in this particular QTL using qRT-PCR technique in both tetep and HP2216 rice lines (Richa et al., 2016). It was found that out of the 11 genes, the gene present on the locus LOC_Os11g47510 was highly expressed after 36 hpi with *R. solani* in rice line tetep as compared to the HP2216. *In vitro* assays confirmed chitinase activity of LOC_Os11g47510 protein as it showed antifungal activity against *R. solani*. Therefore, *LOC_Os11g47510* gene was selected as a candidate gene for cloning and genetic transformation into the sheath blight susceptible rice lineTP309.

In the present investigation, chitinase gene *LOC_Os11g47510* was cloned and transferred into the susceptible rice variety Taipei 309. Putative transgenic plants were characterized at molecular level using PCR and Southern blot tools for gene integration and copy number analysis, whereas qRT-PCR was performed to study the expression of integrated gene in transgenic TP309 rice plants. Among the transgenic TP309 plants subjected to expression analysis, expression of the *LOC_Os11g47510* transcript varied from 3.7- to 9.5-folds higher as compared to the control NT plants. This variation in transgene expression, though under the control of constitutive 35S promoter can be attributed to the site of transgene integration in different events. Effect of site of transgene integration into a euchromatin region on the expression level of the corresponding gene has been reviewed by Kohli et al. (2006). Antifungal bioassay by using detached leaves of transgenic and NT rice plants showed that infection process culminating in lesion development takes place much earlier in NT-control plants as compared to the transgenic plants. Further into the infection process, characteristic necrotic browning feature around the site of lesion was observed in both the transgenic and NT plants after 48–72 hpi, however, the area covered by necrotic regions was more in NT-control plants as compared to the transgenic plants. Formation of infection cushions during early stages of infection has been reported by various researchers. Singh et al. (2003) reported early formation of infection cushion in rice leaf lamina as compared to the leaf sheath. We analyzed the inhibition of fungal mycelia inside the leaf tissue in both, the transgenic as well as NT rice plants inoculated with *R. solani* using light microscope. At 24 hpi, we observed branching of *R. solani* hyphae and colonization along with formation of infection cushion in NT plant, whereas in transgenic plant, very less growth of fungal hyphae was recorded. At 48 hpi, infection was observed in both NT as well as transgenic plant but the number of infection cushions was more in NT plant. Similarly, at 72 hpi the number of infection cushions was much more in NT plant than transgenic plants. Armentrout and Downer (1987) reported the formation of infection cushion at 21 hpi in cotton seedlings and in excised hypocotyls. Infection cushion plays an important role in disease development and its severity as it helps in enzymatic degradation and hyphal penetration inside the leaf tissue (Anderson, 1982; Ghosh et al., 2016; Zhang et al., 2016).

Once inside the plant tissues, the total fungal biomass is also an indicator of the response of the plants against invading fungus. Weihmann et al. (2016) demonstrated the quantification of fungal biomass in maize plants inoculated with *Colletotrichum graminicola*. In the present study, fungal biomass was quantified in the transgenic and NT leaves challenged with *R. solani.* We observed an increased amount of fungal DNA over different time intervals, i.e., 0, 24, 48, and 72 hpi, both in NT and transgenic plants; however, the fungal DNA was much higher in NT plant as compare to the transgenic plant. Therefore, these molecular, phenotypic and microscopic observations can be directly attributed to the transgene overexpression, as validated through qRT-PCR analysis in transgenic TP309 plants. Resistance attributed to overexpression of chitinase gene was also supported by other researchers. Yuan et al. (2004) overexpressed rice chitinase gene (RC24) in rice and reported that the number of lesions was more in non-transgenic rice line as compared to the transgenic line; however, both transformed as well as non-transformed lines could show characteristic necrotic browning features after 2 days of inoculation. In another report, Nandakumar et al. (2007) also observed fewer lesions, i.e., 0–3 in transgenic rice lines as compare to the NT ones, i.e., 4–5, registering more resistance as compare to the NT ones. Further correlating the relation between chitinase expression and lesion length in rice, Shrestha et al. (2008) found a negative correlation between percentage of relative lesion length and chitinase activity in rice. *alit* has also been reported that there was a negative correlation between the mycelial growth of *R. solani* and chitinase activity in the antifungal assay conducted with recombinant LOC_Os11g47510, chitinase protein (Richa et al., 2016). Very recently, Rajesh et al. (2016) analyzed the sheath blight resistance in transgenic ASD16 rice lines expressing a rice *chi11* gene encoding chitinase and found that transgenic line ASD16-4-1-1 gave a better protection against the sheath blight pathogen than the other two lines.

In the present study, the detached leaf assay and real-time qRT-PCR study revealed negative correlation between chitinase expression and number of lesions formed and lesion length caused by *R. solani* and further the chitinase gene overexpression in transgenic plants correlate directly with sheath blight resistance in otherwise susceptible rice line TP309. Usefulness of the detached leaf bioassay for initial screening of the putative transformants for ShB resistance was also supported by qRT-PCR results. It was observed that transgenic events which were showing less disease progression during leaf assay showed high expression of transgene during expression analysis studies. We found that for initial screening of large number of putative transformants, detached leaf assay is a better approach and transformants exhibiting better resistance at initial level needs to be assessed further by expression studies. Therefore, the findings of the present investigation demonstrated that the overexpression of novel chitinase gene from indica rice line can effectively enhance resistance response against *R. solani* infection by inhibiting the growth and branching of hyphae in transgenic plants. Our results are also helpful in enhancing the knowledge and resources available for effective management of rice ShB and the candidate gene validated here can be used for resistance breeding of elite rice lines using molecular breeding or transgenic approach.

## Author Contributions

Conceived and designed the experiments: TS. Performed the experiments: KR and IT. Analyzed the data: KR and IT. Wrote the paper: TS, KR, BD, IT, VS, and JB.

## Conflict of Interest Statement

The authors declare that the research was conducted in the absence of any commercial or financial relationships that could be construed as a potential conflict of interest.

## Footnotes

1 multalin.toulouse.inra.fr/multalin/
