# Supplementary material for: Novel Chitinase Gene LOC_Os11g47510 from Indica Rice Tetep Provides Enhanced Resistance against Sheath Blight Pathogen Rhizoctonia solani in Rice
Source: Front Plant Sci. 2017 Apr 25;8:596. doi: 10.3389/fpls.2017.00596 (PMC5403933; doi:10.3389/fpls.2017.00596)
Supplement: Supplementary file 1 [file Table_1.DOC]

**Supplementary Table 1.** List of primers used in the present study.

| **Primer_id** | **Primer’s Sequence 5’-3’** | **Tm** |
| --- | --- | --- |
| **Development of plant transformation vector** |  |  |
| LOC_Os11g47510 *_*F | TGGATCCATGGCGTCCCAACGCCGGCGAT | 58 |
| LOC_O11g47510 _R | TTCTAGATTAAGCCCAGTGCTTGACATATCTTCCGTAGTGA | 58 |
| Hygromycin _F | ATACCGCGGACAGGCAGCAACGCTCTG | 58 |
| Hygromycin _R | ATACCGCGGCACGACACTCTCGTCTAC | 58 |
| pRT100_CaMv 35S_F | ACCCGGGAAGCTTGCATGCCTG | 58 |
| pRT100_*nos*_R | ACCCGGGAAGCTTGCATGCCTG | 58 |
| **Southern Hybridization** |  |  |
| Hygromycin_F | CACGCCATGTAGTGTATTGACC | 58 |
| Hygromycin_R  **Real-time PCR** | AGAGCCTGACCTATTGCATCTC | 58 |
| LOC_Os11g47510.F | CTCCTCTCCTTGTTAGCGGTGTTCTT | 58.32 |
| LOC_Os11g47510.R | GGAAGGAGATGATGACGATGTTGTA | 54.51 |
| 18SrRNA_F | CTACGTCCCTGCCCTTTGTACA | 63.0 |
| 18SrRNA_R | ACACTTCACCGGACCATTCAA | 62.0 |
| **qPCR for fungal biomass** |  |  |
| RPMK_F | GCAATTGACATTTGGAGTGTGGGCTGCGTAC | 61.0 |
| RPMK_R | CGAGTTGATAGCATAAAAGTCGTCCAGTGA | 61.0 |
| pUC19_F | CGCCAGGGTTTTCCCAGTCACGAC | 61.0 |
| pUC19_R | TCACACAGGAAACAGCTATGAC | 61.0 |
