# Supplementary material for: Novel Chitinase Gene LOC_Os11g47510 from Indica Rice Tetep Provides Enhanced Resistance against Sheath Blight Pathogen Rhizoctonia solani in Rice
Source: Front Plant Sci. 2017 Apr 25;8:596. doi: 10.3389/fpls.2017.00596 (PMC5403933; doi:10.3389/fpls.2017.00596)
Supplement: Supplementary file 2 [file Table_2.DOC]

**Supplementary Table 2.** Genetic transformation of rice with *LOC_Os11g47510* gene and regeneration frequency.

| **Batch No.** | **No. of bombarded calli** | **No. of calli after 1^st^ selection** | **No. of calli after 2^nd^ selection** | **No. of calli after 3^rd^ selection** | **No. of calli regenerated** | **No. of plants regenerated** | **Regeneration efficiency** | **Transformation efficiency** |
| --- | --- | --- | --- | --- | --- | --- | --- | --- |
| Batch1 | 428 | 264 | 198 | 148 | 85 | 5 | 57.4% | 5.8% |
| Batch2 | 400 | 223 | 152 | 112 | 59 | 4 | 52.6% | 6.7% |
| **Total** | **828** | **487** | **350** | **260** | **144** | **9** | **55.3%** | **6.25%** |
