# Supplementary figures and images for: Novel Chitinase Gene LOC_Os11g47510 from Indica Rice Tetep Provides Enhanced Resistance against Sheath Blight Pathogen Rhizoctonia solani in Rice
Source: Front Plant Sci. 2017 Apr 25;8:596. doi: 10.3389/fpls.2017.00596 (PMC5403933; doi:10.3389/fpls.2017.00596)

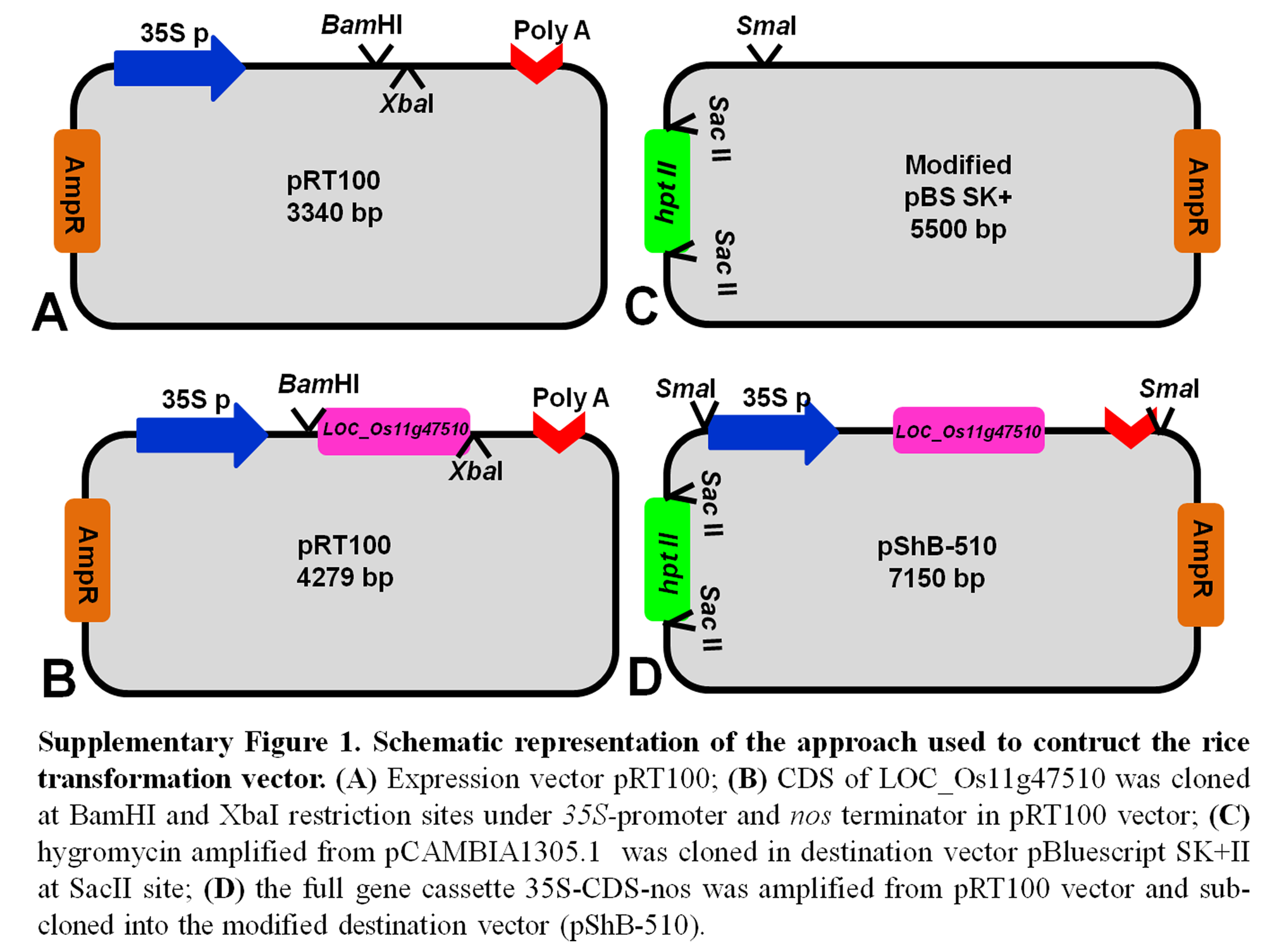

Supplement: Supplementary file 3 [file Image_1.TIF]

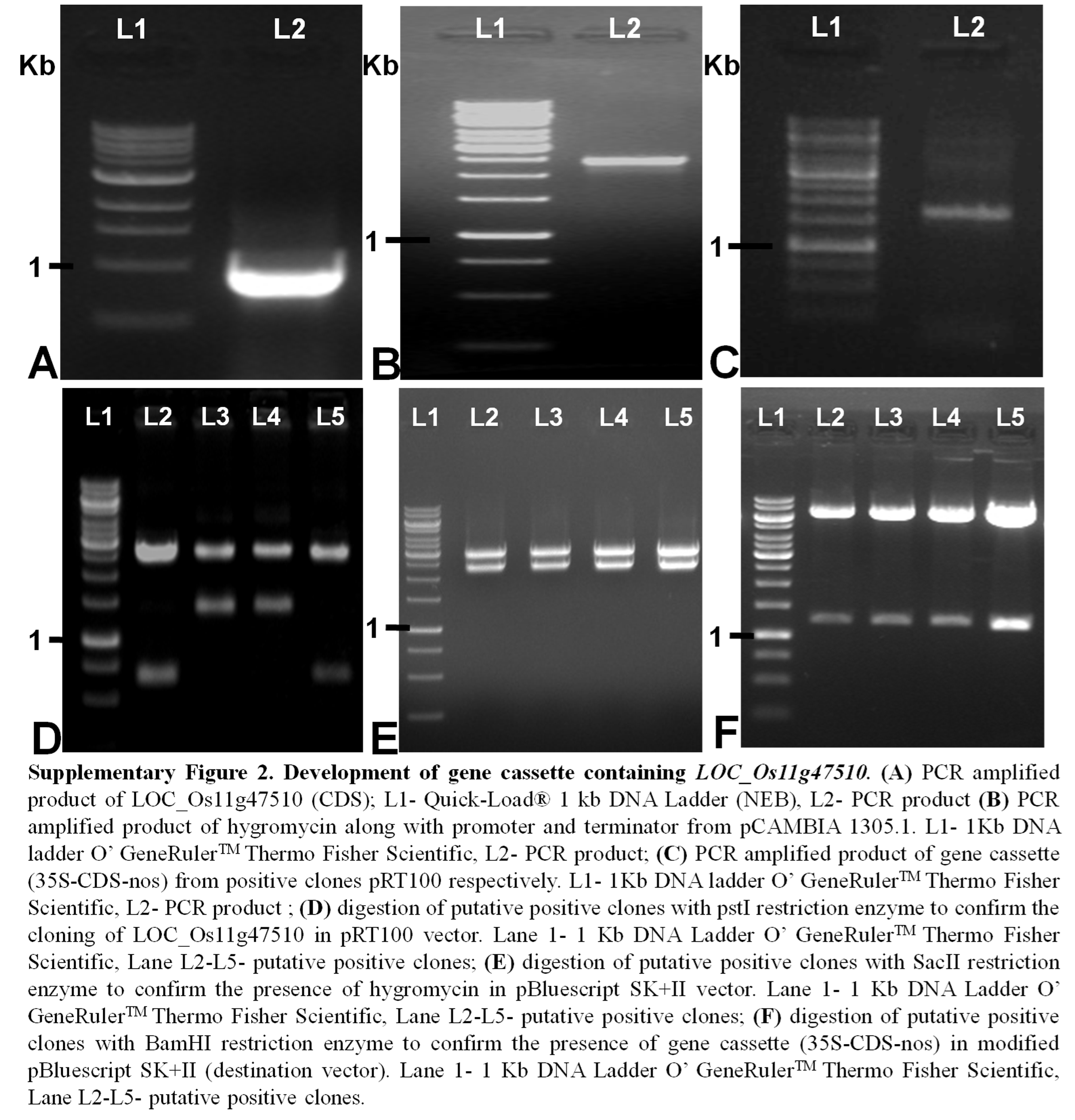

Supplement: Supplementary file 4 [file Image_2.TIF]

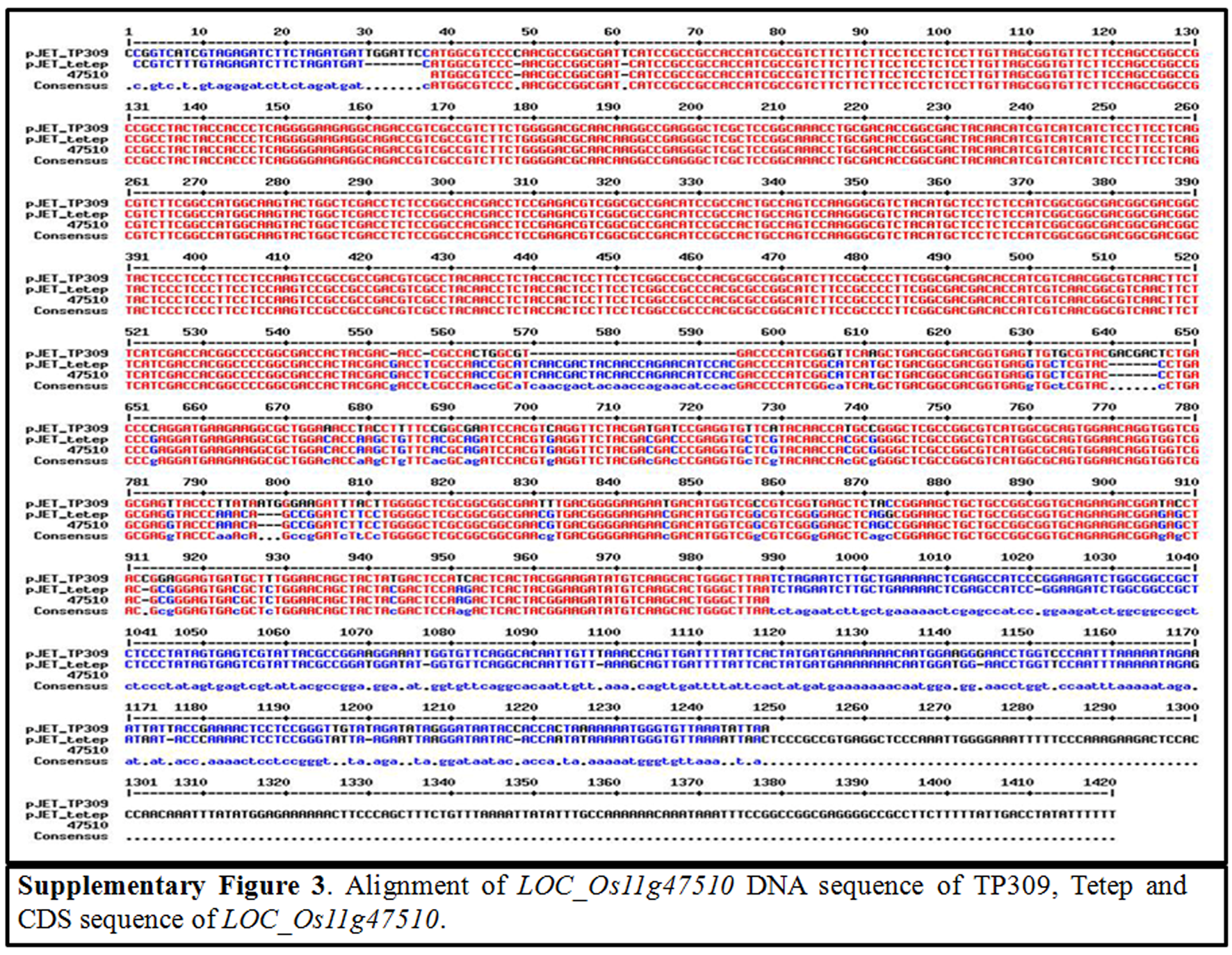

Supplement: Supplementary file 5 [file Image_3.TIF]

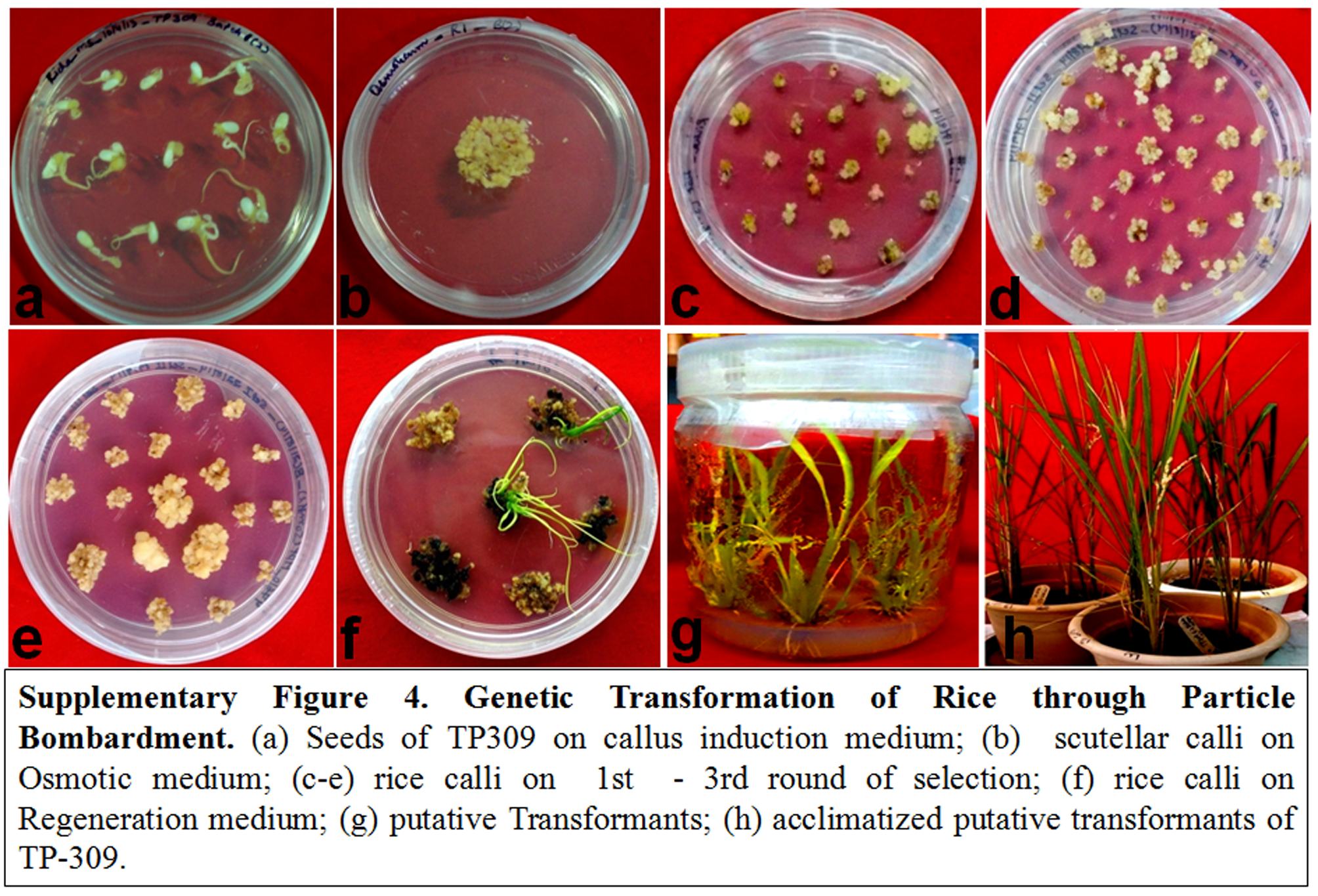

Supplement: Supplementary file 6 [file Image_4.TIF]

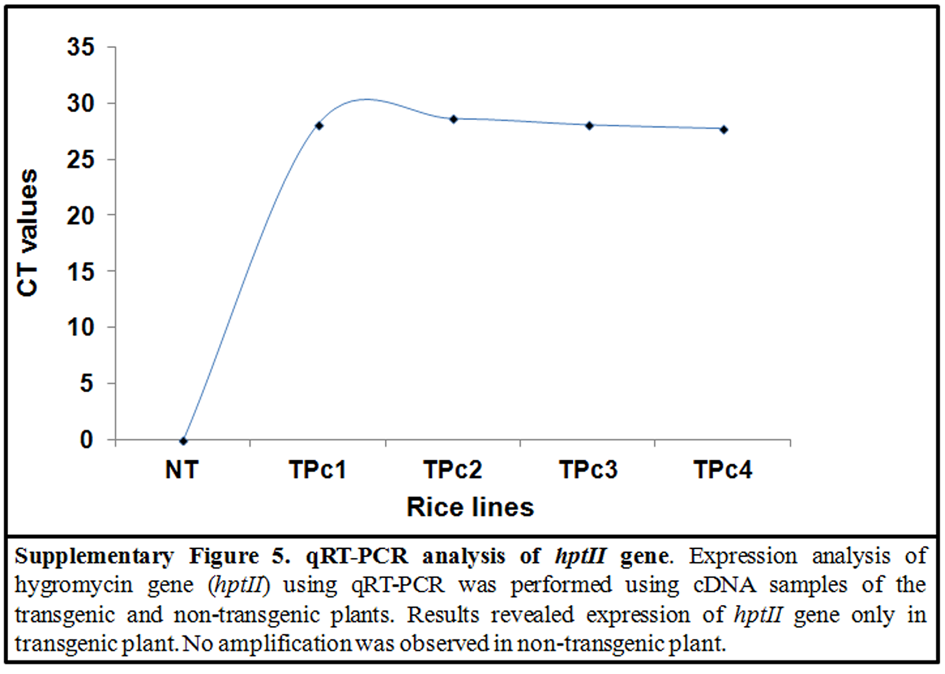

Supplement: Supplementary file 7 [file Image_5.TIF]

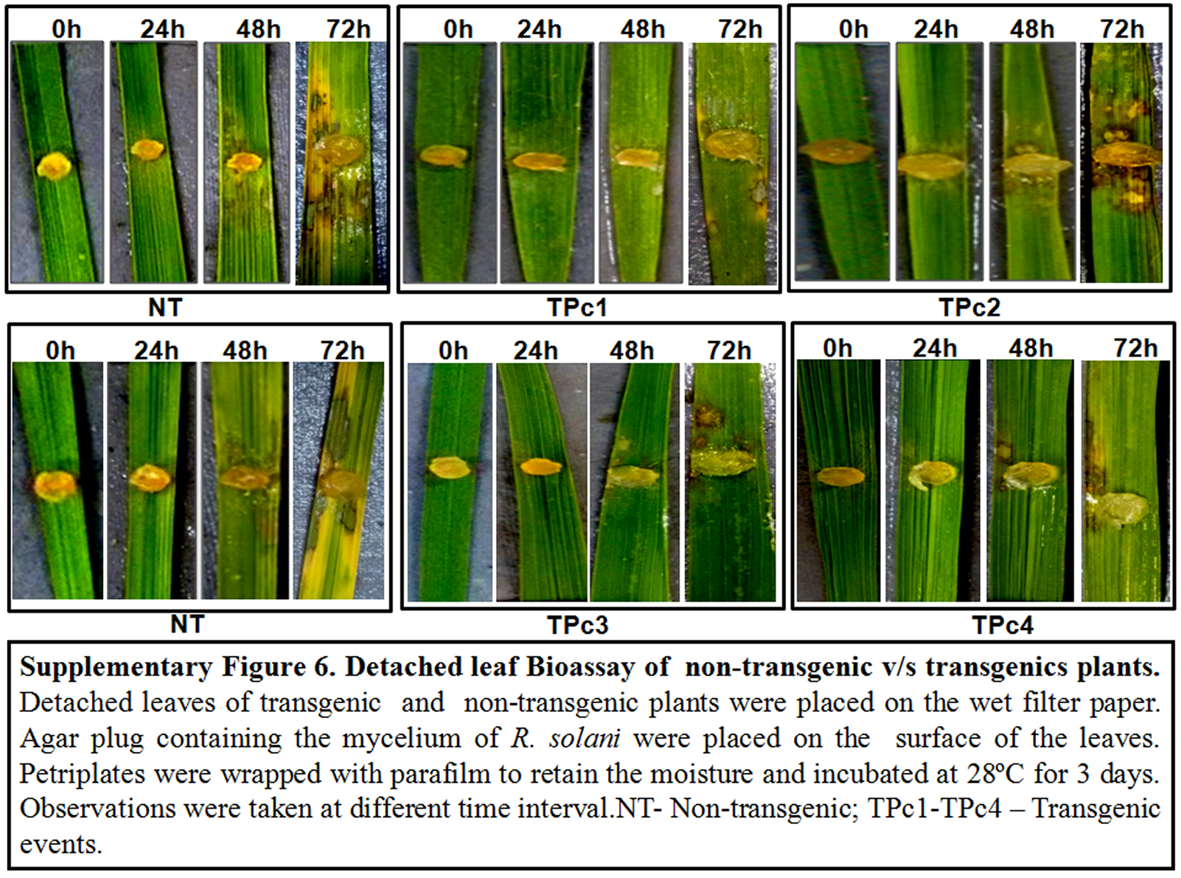

Supplement: Supplementary file 8 [file Image_6.TIF]

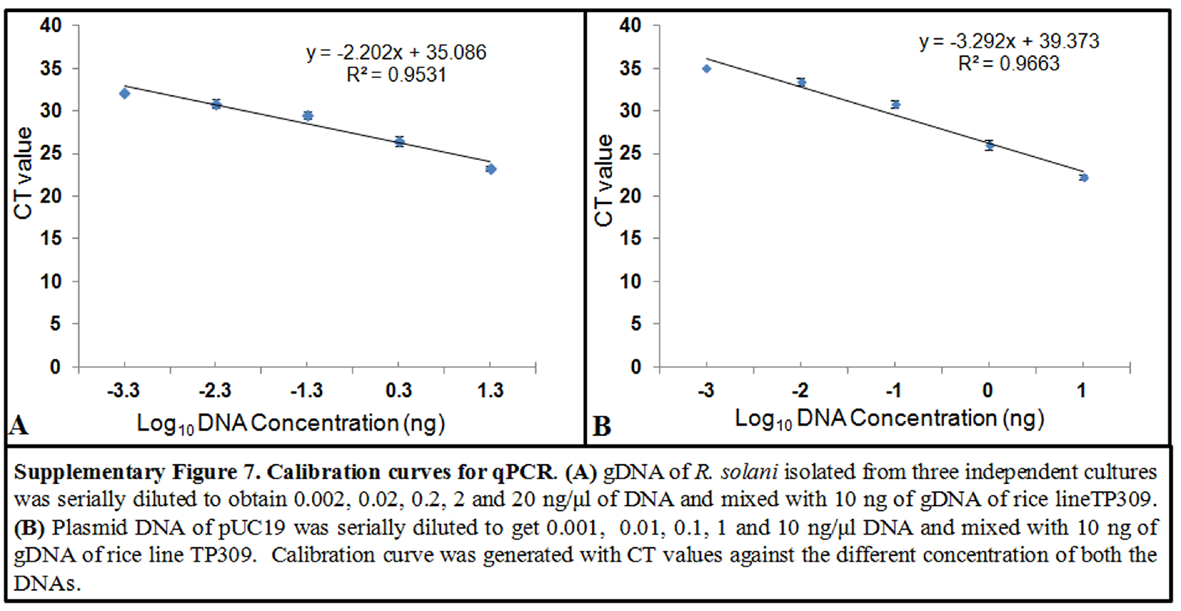

Supplement: Supplementary file 9 [file Image_7.TIF]
